# Single-cell detection of primary transcripts, their genomic loci and nuclear factors by 3D immuno-RNA/DNA FISH in T cells

**DOI:** 10.3389/fimmu.2023.1156077

**Published:** 2023-05-04

**Authors:** Eralda Salataj, Charalampos G. Spilianakis, Julie Chaumeil

**Affiliations:** ^1^ Université Paris Cité, Institut Cochin, INSERM, CNRS, Paris, France; ^2^ Institute of Molecular Biology and Biotechnology-Foundation for Research and Technology Hellas, Heraklion, Crete, Greece; ^3^ Department of Biology, University of Crete, Heraklion, Crete, Greece

**Keywords:** 3D DNA FISH, 3D RNA FISH, immunofluorescence, genome organization, nuclear organization, T cells, thymocyte development, lymphocytes

## Abstract

Over the past decades, it has become increasingly clear that higher order chromatin folding and organization within the nucleus is involved in the regulation of genome activity and serves as an additional epigenetic mechanism that modulates cellular functions and gene expression programs in diverse biological processes. In particular, dynamic allelic interactions and nuclear locations can be of functional importance during the process of lymphoid differentiation and the regulation of immune responses. Analyses of the proximity between chromatin and/or nuclear regions can be performed on populations of cells with high-throughput sequencing approaches such as chromatin conformation capture (“3C”-based) or DNA adenine methyltransferase identification (DamID) methods, or, in individual cells, by the simultaneous visualization of genomic loci, their primary transcripts and nuclear compartments within the 3-dimensional nuclear space using Fluorescence *In Situ* Hybridization (FISH) and immunostaining. Here, we present a detailed protocol to simultaneously detect nascent RNA transcripts (3D RNA FISH), their genomic loci (3D DNA FISH) and/or their chromosome territories (CT paint DNA FISH) combined with the antibody-based detection of various nuclear factors (immunofluorescence). We delineate the application and effectiveness of this robust and reproducible protocol in several murine T lymphocyte subtypes (from differentiating thymic T cells, to activated splenic and peripheral T cells) as well as other murine cells, including embryonic stem cells, B cells, megakaryocytes and macrophages.

## 1 Introduction

High order chromatin folding as well as its organization within the nuclear space are considered as an important regulator of gene expression and nuclear functions. In particular, during lymphopoiesis, spatial nuclear repositioning of different immune genes can be directly linked to their expression while deregulation of the chromatin architecture can be associated with immune-related diseases and cancer (1–9). T-cell lineage specification remains a powerful developmental model system to study *cis*- or *trans*- genomic interactions and gene relocalization, since in most cases these movements can be linked to an immunological response (3, 10). A plethora of studies have reported drastic structural and organizational changes in CD4^+^ cells during the transition from the quiescent state until they encounter T cell receptor (TCR)-specific antigens, which leads to global chromatin remodeling and clonal expansion of the peripheral CD4^+^ cells (11–14). Chromatin rewiring during T cell activation has been documented using either ligation-based chromosome conformation capture (3C) methodologies (population-averaged detection of proximity events between chromatin fragments at the genome-wide level) or imaging-based approaches (such as Fluorescence *in situ* hybridization (FISH) and immunofluorescence) that allow direct visualization of distances between loci and/or nuclear compartments at the single cell level (15). Despite the fact that during the recent years, the rapidly evolving field of chromatin organization has benefited from the use of ligation-free sequencing methodologies, these methodologies have not been applied yet in immune cells (16–18).

Several groups have reported how chromosomal interactions between distal regulatory DNA sequences (such as promoters, enhancers, insulators, locus control regions (LCR) and non-coding sequences (CNSs)) play a crucial role during T cell differentiation and are directly correlated to gene expression levels (3, 19–21). Moreover, during thymocyte maturation and naïve T cell development, 3-dimensional DNA FISH experiments have documented that differential associations of genes, subnuclear chromatin reorganization and cell type specific-gene repositioning relative to permissive or repressive domains is linked to transcriptional changes and alternative cell fate decisions in CD4^+^ and CD8^+^ cells (22, 23). Furthermore, imaging-based approaches and 4C experiments have dissected in detail the dynamics of conformation and organization of antigen-receptor loci (contraction/decontraction, relocalization to nuclear sub-compartments such as nuclear lamina or pericentric heterochromatin (PCH), intra- and inter-allelic pairing) during V(D)J recombination in lymphocytes (24–33). Moreover, 3D RNA/DNA FISH have revealed how upon TCR activation, activated helper T cells increase their global transcription levels with the remodeling of their chromatin accessibility, leading to dramatic enlargement of their nuclei and rewiring of the 3D architecture, with cell type-specific genes being repositioned (34–37).

3D DNA FISH is a powerful molecular and cytological methodology to map gene loci within the interphase nucleus, or on mitotic chromosomes and was firstly introduced by Rudkin and Stollar in 1977 (38). Since then, FISH has been broadly used in many research and clinical applications, such as diagnosis of various genetic diseases or cancer, including identification of chromosomal rearrangements (deletions, amplifications and translocations), changes in ploidy or identification of novel oncogenes in various hematologic and solid tumors (39). Over the past decades FISH has undergone substantial development accompanied by remarkable technical advances which in combination with high-resolution microscopy studies of chromatin structure, has led to the better understanding of 3D chromatin organization (40–45). Despite the constantly expanding arsenal of the C-derived methodologies to capture chromatin interactions at a genome-wide level (46), 3D DNA FISH still remains one of the most accurate methodologies that can provide quantitative measurements for the position of genes within single nuclei, with respect to their chromosome territories and/or with respect to nuclear compartments, as well as distances between several genomic loci (15).

3D RNA/DNA FISH enables studying the three-dimensional organization of the genome and/or its transcripts at the single cell level in individual cells, but also in tissue sections or whole organisms (47). Depending on the protocol, FISH variants can detect either DNA (the physical location of a gene locus) or RNA (detection of a primary transcript). In cases where the location of gene activity (positioning relative to subnuclear landmarks) needs to be mapped, then a combination of both methodologies is required, leading to detection of the precise location of the gene locus (3D DNA FISH) together with the primary gene transcript (3D RNA FISH). In interphase nuclei, 3D DNA FISH can map precisely either an entire chromosome territory (CT), part or sub-region of a chromosome or individual or multiples gene physical localizations, depending on the type and length of the probes. Measurements can be performed to assess gene interactions (*cis*- and *trans*-), or the location of a gene with respect to its chromosome territory. 3D RNA FISH allows the detection of RNAs and the dissection of the mechanisms of gene expression, especially when combined with other methodologies such as genome editing (48–53). Combinations of 3D DNA FISH (from gene-specific probes to entire chromosome paints), 3D RNA FISH, and immunostaining of nuclear factors allow the better understanding of 3D chromatin organization within the nuclear space, by identifying not only the localization of genes but also important subnuclear landmarks such as chromosome territories, particular chromatin domains (speckles and paraspeckles, the nucleolus, or the nuclear lamina).

Here we present a detailed end-to-end 3D immuno-RNA/DNA FISH protocol to study multiple levels of chromatin organization during T cell development. Our protocol provides reliable results of high-quality bright signal, low background staining and can be applied to several T cell subtypes. The protocol presented here describes the methodology for combinations of immunofluorescence with RNA and/or DNA FISH in mouse embryonic stem cells (mESC) (54–57), whole thymocytes, naïve and differentiating T and B lymphocytes (25–27, 58–61), activated T and B cells (60, 62) and leukemic cell lines (63). In our hands, the following protocol is also successfully working in both murine and human primary and immortalized cell lines such as HEK293T, RAW264.7 and mouse embryonic fibroblasts (MEFs). Although we have already published the protocols for the methodologies regarding 3D immuno-RNA FISH, 3D immuno-DNA FISH and 3D RNA/DNA FISH (55, 57, 58), the current protocol represents a step forward with the combination of the three stainings together (ie. 3D immuno-RNA/DNA FISH) including also chromosome painting.

## 2 Materials and equipment

All equipment and reagents required for the protocol described here are listed in **Table 1**. The composition of buffers and solutions needed for 3D immuno-RNA/DNA FISH, are presented in **Tables 2**, **3**.

**Table 1:** Reagents and resources needed for 3D immuno-RNA/DNA FISH.

Reagents and resources needed for 3D immuno-RNA/DNA FISH	PROVIDER	REFERENCE
10x PBS	Sigma-Aldrich (MERCK)	D1408
20x SSC	Sigma-Aldrich (MERCK)	S6639
Aminoallyl-dUTP-ATTO-488, 1mM	Euromedex	NU-803-XX-488-L
Aminoallyl-dUTP-ATTO-550, 1mM	Euromedex	NU-803-XX-550-L
Aminoallyl-dUTP-ATTO-647N, 1mM	Euromedex	NU-803-XX-647N-L
BSA Fraction V, 7.5%	Gibco^TM^ (Thermo Fisher Scientific)	15260037
BSA, 20mg/ml	NEB	B9000S
Chloramphenicol, powder	Sigma-Aldrich (MERCK)	C7795
DAPI, powder	Sigma-Aldrich (MERCK)	D9542
DEPC	Thermo Fisher Scientific	J14710.AC
Dextran sulfate, powder	Sigma-Aldrich (MERCK)	D8906
DH5a E. coli competent bacteria	NEB	C2987H
EDTA, pH8, powder	Sigma-Aldrich (MERCK)	EDS
Ethanol, absolute	Sigma-Aldrich (MERCK)	51976
Fibronectin, 1mg/ml	Sigma-Aldrich (MERCK)	FC010
Formaldehyde, 16% solution	Thermo Fisher Scientific	28908
Formamide, 100%	Sigma-Aldrich (MERCK)	47671
Gelatin, 0.1% solution	Millipore (MERCK)	ES-006-B
Goat-anti rabbit antibody AlexaFluor 680 (GAR-680; dilution 1/1000)	Thermo Fisher Scientific	A21109
Goat serum	Eurobio	S-1000
H_2_0 sterile	Sigma-Aldrich (MERCK)	W3500
Human Cot-1 DNA, 1mg/ml	Invitrogen^TM^ (Thermo Fisher Scientific)	15279011
Hydrochloric acid (HCl), 37% solution	VWR	20252
LaminB1 antibody (rabbit polyclonal; dilution 1/1000)	Abcam	ab16048
Mouse Cot-1 DNA, 1mg/ml	Invitrogen^TM^ (Thermo Fisher Scientific)	18440016
Phenol-chlorophorm-Isoamyl alcohol solution	Sigma-Aldrich (MERCK)	P2069
Poly-D-Lysine, 0.1mg/ml solution	Gibco^TM^ (Thermo Fisher Scientific)	A3890401
Poly-L-Lysine, 0.1% solution	Sigma-Aldrich (MERCK)	P8920
Potassium Acetate (KoAc), pH 5.5, 3M	Invitrogen^TM^ (Thermo Fisher Scientific)	AM9610
Prolong Diamond antifade reagent	Invitrogen^TM^ (Thermo Fisher Scientific)	P36965
Purelink PCR purification kit	Invitrogen^TM^ (Thermo Fisher Scientific)	K31001
RNAse A, 10mg/ml	Thermo Fisher Scientific	EN0531
RNAse Inhibitor (Ambion), 40U/ml	Invitrogen^TM^ (Thermo Fisher Scientific)	AM2682
RVC (Ribonucleoside Vanadyl Complex), 200mM	NEB	S1402S
Salmon Sperm, 10mg/ml	Invitrogen^TM^ (Thermo Fisher Scientific)	15632011
SDS, powder	Invitrogen^TM^ (Thermo Fisher Scientific)	15525017
Sodium Acetate (NaAc), pH 5.2, 3M	Sigma-Aldrich (MERCK)	S7899
Sodium Hydroxyde (NaOH), 1N	Sigma-Aldrich (MERCK)	S2770
Topo TA cloning kit	Invitrogen^TM^ (Thermo Fisher Scientific)	450641
Tris, powder	Sigma-Aldrich (MERCK)	T6791
Triton-X-100	Euromedex	2000-C
TSA Biotin System	Perkin Elmer	NEL700A001K
Tween-20	Euromedex	2001-C
Vysis Nick translation kit	Abbott Molecular	07J00-001
EQUIPMENT	PROVIDER	REFERENCE
1.5ml tube	Eppendorf	–
15ml tube	Falcon	–
24-well plate	Techno Plastic Products AG	92024
50ml tube	Falcon	–
6-well plate	Techno Plastic Products AG	92006
Coverslip, circular (12mm diameter)	Paul Marienfeld GmbH	111520
Coverslip, square (22x22mm)	Paul Marienfeld GmbH	107052
Dark and humid hybridization chamber	N/A	–
Dark Eppendorf tubes	Sarstedt	72690004
Forceps Dumont #5	Fine Science Tools	11251-10
Glass slides, superfrost	VWR	631-0705
Hybridization oven Shake 'N' Bake	Boekel Scientific	136400
Orbital shaking table (Titramax 101)	Heidolph Instruments	544-11300-00
Refrigerated microcentrifuge for 1.5/2ml tubes	N/A	–
Tabletop centrifuge	N/A	–
Thermomixer C (preferred) or waterbath set at 16°C (for nick translation) and heatblock up to 75°C (for probe preparation and probe/slide denaturation)	Eppendorf	5382000015
Vortex	N/A	–
Water bath up to 80°C	N/A	–
Whatman paper	N/A	–

**Table 2:** Buffers and solutions needed for cell fixation and probe generation.

Buffers needed for 3D immuno-RNA/DNA FISH (*freshly prepared*)	
2x SSC	
1x PBS	
70% and 100% ethanol	
Fixation solution:	2% Formaldehyde, 1xPBS, pH 7.0-7.4 (adjust pH with HCl if necessary)
Permeabilization solution:	0.5% Triton X-100, 1xPBS, pH 7.0-7.4
Blocking Solution	5% BSA, 1x PBS
Alternative Blocking solution:	1% BSA, 10% natural Goat serum, 0.1% Tween-20, 1x PBS
Post-fixation solution:	0.7% Triton-X, 0.1M HCl
Denaturation solution (*for Option 2 of DNA FISH*):	50% Formamide, 2x SSC, pH 7.0-7.4 (adjust pH)
Washes after RNA/DNA FISH:	

**Table 3:** 2x Hybridization buffer.

2x Hybridization buffer (can be stored at -20°C for months)	
Final concentration:	Recipe for 1ml:
20% Dextran Sulfate	400ml of 50% dextran sulfate
4x SSC	200ml of 20x SSC
4mg/ml BSA	200ml of 20mg/ml BSA
20mM Ribonucleoside Vanadyl Complex (RVC)	100ml of 200mM RVC
	100ml H_2_O

## 3 Methods

**Figure 1:**
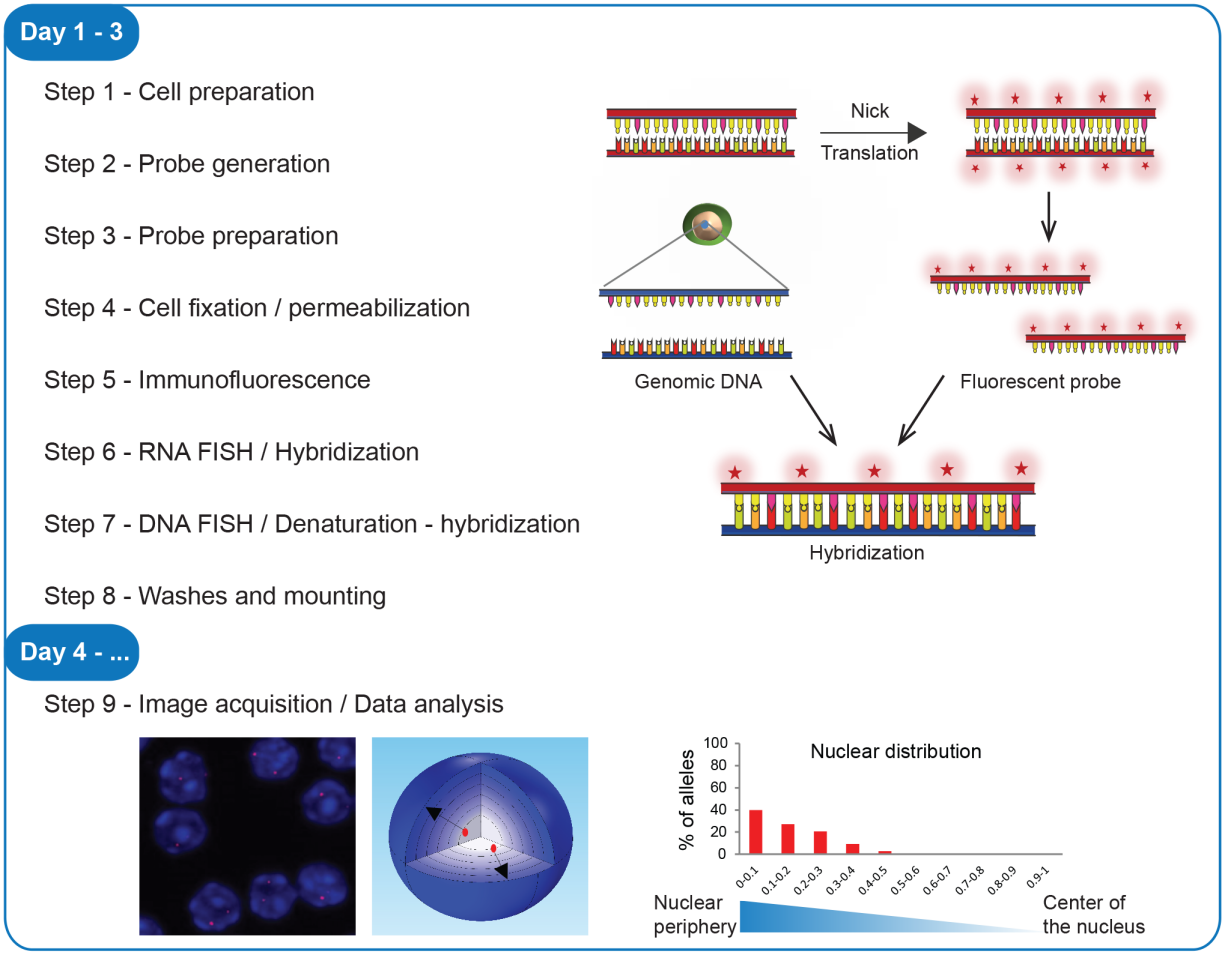
Workflow of 3D immuno-RNA/DNA FISH. Steps of the experimental procedure, from the cell preparation to image acquisition and data analysis with a scheme of the fluorescent probe labeling, hybridization and examples of image analyses.

For a better experimental design, prior to implementing the 3D immuno-RNA/DNA FISH protocol described here, refer to **Figure 1** to choose the version best suited for your experiment. As presented below, in this protocol both cell and probe preparations are conducted in advance before the stepwise procedure part. All solutions necessary for the step-by-step procedure must be sterilized and RNase/DNase free.

### 3.1 Cell preparation

#### 3.1.1 Adherent cells

Glass coverslips (diameter from 12mm to 22mm and thickness ~1μm so the 3D structure of the cells during the hybridization step is not disrupted) are placed into a 24- or 6-well plate respectively and sterilized either using a UV-lamp for 30 minutes or rinsed in 100% ethanol and dried before use. Coverslips are then coated with 0.1% gelatin to allow cell adhesion. Briefly, coverslips are incubated in a 0.1% gelatin/1x PBS solution at 37°C for a minimum of 1 hour, and the solution is carefully aspirated just before use. Alternatively for adherent cells that may not attach on 0,1% gelatin, coverslips can be coated with 10μg/ml Fibronectin/1x PBS at 37°C for a minimum of 2 hours and rinsed three times with 1x PBS just before use. Cells are then plated onto the coated coverslips and cultured for at least 24 hours until they reach a ~60-70% confluency. Cells are then fixed (see section 3.4).

#### 3.1.2 Non-adherent cells

**Figure 2:**
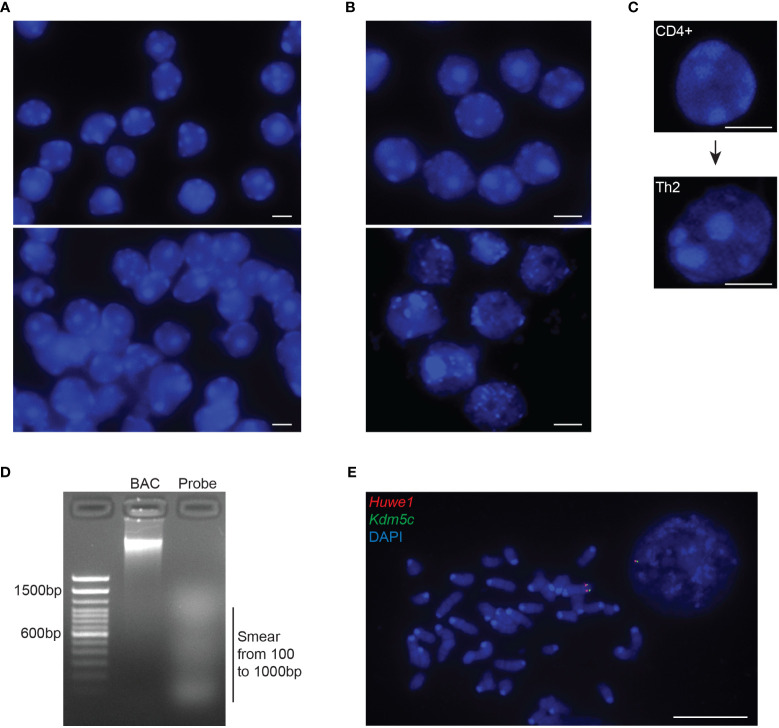
Quality controls of intact 3D nuclei and probe preparation. **(A)** Representative images of mouse DP T cells depicting nuclei with adequate confluency (top panel) or over-confluency (bottom panel). Adjacent or overlapping nuclei will impair the segmentation of individual nuclei in order for the 3D imaging softwares to normalize distance calculations through the diameter or the volume of each nucleus. **(B)** Representative images of nuclei with preserved (top panel) or disrupted 3D structure (bottom panel). Disruption of the 3D architecture (dehydrated, swollen or exploded nuclei) during cell preparation will affect the analysis of the gene position and allelic distances within the 3D nuclear space. **(C)** Example of the increased nuclear volume of naïve CD4^+^ cells upon TCR activation and differentiation into T helper 2 cell. **(D)** Representative gel image depicting the ideal smear of the BAC fragments constituting a fluorescent probe for RNA/DNA FISH during the Nick Translation procedure (NT). The optimal NT fragments are 100–1000 nucleotides long. **(E)** Representative metaphase spread from mouse embryonic stem cells, using the NT generated DNA probes from a mouse BAC, RP24-157H12 (spanning the *Huwe1* gene, labelled in Atto-dUTP 550, red) and a mouse fosmid, WI1-88O7 (spanning the *Kdm5c* gene, labelled in Atto-dUTP 488, green) both located in chromosome X. Nuclei and metaphase spread are counterstained with DAPI (blue). Scale bar = 5µm.

Non-adherent cells are grown in culture or directly freshly FACS-sorted. Glass coverslips are coated with poly-L-lysine. Briefly, coverslips are incubated in a 0.1% poly-L-lysine solution for a minimum of 15 minutes, air-dried and placed into 24- or 6-well plates. A 0.1mg/ml poly-D-Lysine solution can alternatively be used in cases where cells are not attaching with poly-L-lysine. In our lab we successfully used poly-L-lysine for mESC and T cell subtypes, while for naïve and activated B cells poly-D-Lysine may be recommended. Cells are then dropped onto the coated coverslips (~3x10^5^ cells in a 30µl drop of 1X PBS) and left to sediment for about 10 minutes without shaking (at this point cell confluency and seeding should be checked under a microscope). The confluency of the cells plated on glass coverslips should approximately be around 60-70%, cells being intact and not overlapping with each other (**Figure 2A**). A higher confluency might affect the quality of the permeabilization and/or the denaturation of the genomic DNA (thus the quality of the fluorescent signals), the 3D structure of the cells (nuclei), and the 3D measurements for the normalization of distances between the genomic loci. Cells are then fixed (see section 3.4).

### 3.2 Probe generation

#### 3.2.1 Probes generated by Nick Translation

##### 3.2.1.1 DNA FISH probe templates

Fluorescent probes for 3D DNA or RNA FISH can be generated by different approaches. We are mostly using the process of Nick Translation. Different types of vectors carrying genes or genomic regions of interest can be used as templates, from plasmids (>10kb), to fosmids (~50kb) an,d bacterial artificial chromosomes (BACs) (~100-200kb). We are mainly using fosmids and BACs for more robust signals. BACs and fosmids are purchased in the form of bacterial glycerol stocks (from CHORI or Thermo Fisher Scientific) and should thus be amplified and purified. DNA isolation can be performed either with commercially available kits specific for large DNA fragments, or using in-house protocol as described here. Firstly, each clone is streaked onto Petri dish plates with LB media containing 12.5μg/ml chloramphenicol and incubated overnight in a 37°C incubator. A single colony is selected and inoculated into 1L of liquid Luria broth (LB) medium supplemented with 12.5 μg/ml chloramphenicol. After overnight incubation at 37°C, bacteria are spun for 15 minutes at ~4300 RCF at room temperature (RT). The pellet is resuspended in 10 ml ice-cold resuspension solution (50mM Tris pH 8.0, 10mM EDTA pH 8.0 and 100ng/ml RNase A). An equal volume of lysis solution (0.2M NaOH, 1% SDS) is added for a 5-minute cell lysis at RT and neutralized with an equal volume of neutralization solution (3M KAc pH 5.5) for 5 minutes on ice. Upon centrifugation at ~4300 RCF for 30 minutes at RT the supernatant is filtered through a Whatman paper, and the BAC DNA is then precipitated with 2.5 volumes of 100% ethanol. Precipitated DNA is further centrifuged at ~4300 RCF for 45 minutes at 4°C. The DNA pellet is washed once more with 75% ethanol and then resuspended in 200μl ddH_2_0. The next steps include first RNAse A (100ng/ml) treatment of the BAC DNA for 2 hours at 37°C, phenol-chloroform-isoamyl alcohol purification and precipitation with 3M Sodium Acetate (NaAc, 1/10 Volume) and 2.5 volumes of 100% ethanol. After a final centrifugation at ~22.000 RCF for 15 minutes at 4°C, the DNA pellet is briefly air dried and resuspended in 500 μl ddH_2_O. Next, the concentration and the purity of the isolated BAC DNA are determined with both spectrophotometric assay (Nanodrop) and agarose gel electrophoresis. To confirm BAC DNA sequence, PCR reactions with gene-specific primers may be performed as following: initial incubation at 94°C for 5 minutes, 40 cycles of denaturation (94°C for 40 seconds), annealing (60°C for 40 seconds) and extension (72°C for 30 seconds) and a final extension at 72°C for 10 minutes. The PCR products are then analyzed by electrophoresis of the PCR products on 1-1.5% agarose gel depending on the expected product size.

##### 3.2.1.2 RNA FISH probe templates

Plasmids, fosmids or BACs can also be used to generate nick translated RNA FISH probes (as DNA-RNA hybrids) for the visualization of nascent RNA transcripts, when they only contain the transcribing region of interest (56). Alternatively, specific shorter probes (<1-1.5kb long) can be generated by PCR from BACs, fosmids or genomic DNA, in particular for smaller transcripts or small non-coding RNAs such as microRNAs (60). In this case the PCR fragments are subsequently cloned into a TOPO^®^ TA vector, transformed in DH5a *E.coli* competent cells, amplified and isolated by regular mini-preparation of DNA. After reconfirmation of the insert by PCR and/or sequencing, fluorescent probes are generated by Nick Translation of DNA. Small RNA FISH probes of 5 to 15 oligonucleotides, ~500bp long can also be amplified from genomic DNA, purified, amplified and labelled by nick translation (62). RNA FISH probes can also be generated from cDNA, where the cDNA sequence is cloned into a plasmid vector, while the strand specific probes can be prepared with *in vitro* transcription using a viral RNA polymerase promoter of the vector (64).

##### 3.2.1.3 Probe labeling by Nick Translation

Fluorescent probes are labeled by Nick Translation using the Vysis Nick Translation kit (other kits or in house custom-made enzymatic reactions can be used) (58). Each reaction is prepared with 1-2μg of vector DNA, supplemented with fluorescent dUTP (488nm, 550nm or 647nm), following the manufacturer’s instructions. We are routinely using Aminoallyl-Atto-dUTP (Jena Bioscience, Euromedex; see **Table 1**), but we have also successfully used ChromaTide Alexa Fluor dUTP (Thermo Fisher Scientific).

- Mix the reagents as shown in **Table 4** (final volume 40μl), vortex and spin down briefly.
- Add 10μl of NT enzyme and mix gently.
- Incubate overnight at 15°C (use a Thermomixer or a refrigerated water bath).
- Incubate for 10 minutes at -20°C in order to inactivate the enzymatic reaction. At this point probes can be stored in the dark, at -20°C for months.

**Table 4:** Nick translation.

Nick Translation Mix	
Nuclease free H_2_O	(17.5-x) μl
NT buffer	5 μl
0.1mM dTTP	5 μl
0.1mM dNTP mix	10 μl
0.2mM Fluo-dUTP	2.5 μl
DNA template	x μl (~1-2μg)
TOTAL	40 μl

Probe size should be optimized. Probe fragmentation can be reconfirmed *via* agarose gel electrophoresis on a 1.8% gel, displaying an ideal size between 100-500bp which allows for a successful hybridization (**Figure 2D**). A faint smear should be visible between 100 to 1000bp. If the fragmentation of the BAC DNA is not ideal (e.g., bulk of the smear being higher than 500-1000bp) then 3μl of the enzyme mix can be added and the samples should be incubated for an extra 2-4 hours. The smear from the probe fragmentation may not be visible when preparing probes labeled with fluorophores emitting in far red light (Atto-647), while the unincorporated fluorescent dUTP usually appear as a rapidly migrating band.

*Critical Point 1: Before conducting the final 3D DNA FISH experiment in interphase nuclei, we recommend to first check the quality of the 3D DNA FISH probes. A quick and very efficient way, is through the hybridization on metaphase spreads* (65) *(* **Figure 2E** *). Metaphase hybridization is easier than in interphase nuclei where the cells need to be efficiently permeabilized in order to allow for efficient probe penetration into the nucleus. If no 3D DNA FISH signal can be detected in the metaphase samples, then the quantity of probe, needed for efficient DNA hybridization/detection, needs to be further optimized.*

#### 3.2.2 Commercially available RNA and DNA FISH probes

3D DNA FISH probes can also be purchased commercially as labelled or unlabeled customized oligonucleotides targeting specific genomic regions. We have successfully used probes composed of fluorescently-labeled oligonucleotides designed and produced by Roche Nimblegen for both 3D RNA and DNA FISH in mouse double-positive T cells. We used a probe covering the entire *Tcra* locus (~1.8Mb) (26) as well as an ‘exome probe’ covering the gene rich-regions of the 14 chromosome (66). Oligopaints are bioinformatically designed based on the gene sequence and can target regions from few kilobases to many megabases (67). Oligopaints offer barcode flexibility, leading to an increased number of targets from several genes of a whole chromosome (47, 68). Oligopaints can be strand-specific and offer a higher specificity and high resolution of gene of interest. However, in some cases, these hybridizations may show lower intensity signal and/or higher background signal due to the lower number of accumulated fluorophores when compared with NT derived probes.

Probes for chromosome painting covering entire mouse or human chromosomes are commercially available. In our lab we have successfully used chromosome painting probes provided by MetaSystems or Cambio (26, 27, 56, 61). RNA FISH probes can also be commercially available and customized based on the target of interest and microscope’s dye filter set compatibility (Stellaris™, Biosearch Technologies).

### 3.3 Probe preparation [timing: ~2 hours]

*Critical Point 2: all the steps involving fluorescent probes should be performed in the dark in order to avoid photobleaching. Dark Eppendorf could be used during the probe preparation.*

#### 3.3.1 Precipitation of probes generated by Nick Translation

- Use 3-8µl of nick translated probe per coverslip (The amount of probe for optimal labelling may vary between probe/in each cell line). We usually use between 100 and 400 ng/coverslip (the average being 150-200ng) for both murine and human probes.
- Add 2µl of Salmon Sperm DNA/coverslip.
- In order to block repetitive sequences from large probes like BACs or fosmids, that may lead to unspecific background signal, adding 1-5μg of Cot-1 DNA per coverslip (mouse or human, depending on the cell species of interest) is recommended. Amount of Cot-1 DNA used per each hybridization should be optimized depending on the probe. However, it should not be used when the gene/region of interest contains many repetitive DNA sequences, as in this particular case a ‘pre-annealing’ step may inhibit the signal.
- Precipitate with 1/10 volume of 3M Sodium Acetate (NaAc pH 5.2) and 2.5 volume of 100% Ethanol.
- *Example: mix 5μl of nick translated product (probe), 5μl of Cot-1 DNA, 2μl of Salmon Sperm, 88μl of H_2_O and 10μl of NaAc and vortex. Add 250μl of 100% Ethanol and vortex.*
- Spin down for 30 minutes at ~16000 RCF at 4°C.
- Remove the supernatant and add 200 µl of 70% Ethanol.
- Spin down for 5 minutes at ~16000 RCF at 4°C.
- Dry the DNA pellet (use either a SpeedVac vacuum concentrator for 1 minute at RT or incubate at 37°C for 5-10 min).
- Add 5-10µl of deionized formamide per tube without pipetting up and down, as the pellet may get stuck in the tip, and directly incubate in the dark at 37-42°C under agitation (200-300rpm) until complete resuspension. Formamide helps in reducing the hybridization temperature without affecting the 3D structure of the nucleus. The volume of formamide depends on the size of the coverslips used: typically, 5μl of formamide are needed when using small round glass coverslips (12-13mm in diameter), and 10μl of formamide are needed for 18mm round glass and 22x22mm square coverslips.

During probe preparation, several probes can be mixed together. For 3D RNA FISH: different probes labeled with different fluorophores can be coprecipitated together for simultaneous detection in the same cells except if different treatments are required (with or without pre-annealing for example). In the latter case, probes should be precipitated and resuspended separately (in half the volume of formamide each) and pooled just prior to hybridization with the cells. For 3D DNA FISH: several probes labeled with different fluorophores can be precipitated together for simultaneous detection in the same cells. However, in the case of performing 3D DNA FISH following Option 1 (see section 3.7.1) and if probes require different treatments (with or without pre-annealing for example), they should be precipitated and resuspended separately (in half the volume of formamide each) and pooled just prior to the hybridization step with the cells. When mixing RNA and/or 3D DNA FISH probes with chromosome paints, RNA and/or DNA probes should be resuspended in half the volume of formamide, since chromosome paints are ‘ready-to-use’ and already resuspended in a hybridization solution.

*Critical Point 4: In cases where probe precipitation is not successful, it may be replaced with probe purification. In this case, after the Nick Translation reaction, the probe is purified using a PCR purification kit, following the manufacturer’s instructions. 100 ng of DNA probe and 1-5μg mouse Cot-1 DNA are lyophilized using a Concentrator plus (Eppendorf) for 15 minutes at 45°C before resuspension in formamide (see step 3.3.1.j). At this step, we recommend the use of dark Eppendorf tubes in order to protect the photosensitive fluorophores.*

#### 3.3.2 Denaturation/pre-annealing/hybridization of probes

The following steps depend on the protocol of 3D RNA/DNA FISH chosen (see sections 3.6 and 3.7). Thus, follow either section 3.3.2.1 for a combined 3D RNA/DNA FISH protocol or section 3.3.2.2 for only 3D DNA FISH protocol.

##### 3.3.2.1 Option 1: For 3D RNA FISH and 3D DNA FISH (for details see sections 3.6 and 3.7.1, respectively)

- Denature probe(s) for 7 minutes at 75-95°C (use a thermomixer or a heat block). For chromosome paints, denature 5-10μl of ready-to-use chromosome solution at 75°C for 5 minutes (volume should be troubleshooted but we usually use 5μl per coverslip).
- When pre-annealing with Cot-1 DNA, incubate probe for 30-60 minutes at 37°C. The length of incubation depends on each probe: We recommend starting with 30 minutes, but further optimization might be required (increasing/decreasing the time depending on the intensity of background signal). Probes that don’t need pre-annealing (e.g., plasmids, small probes and chromosome paints) can be kept on ice after denaturation until hybridization or denatured just prior to hybridization.
- Mix the different types of probes (DNA, RNA and/or chromosome paints).
- Add 5-10µl of Hybridization buffer (e.g., equal volume of formamide: see step 3.3.1.j) and proceed to overnight hybridization with cells at 37°C (see. section 3.6 for 3D RNA FISH and section 3.7.1 for DNA FISH).

##### 3.3.2.2 Option 2: For 3D DNA FISH (see section 3.7.2 for details)

- Mix the different types of probes if necessary (for example NT probes and chromosome paints).
- Add 5-10µl of Hybridization buffer (e.g., equal volume of formamide: see step 3.3.1.j).
- Denature probe(s) for 3 minutes at 75-95°C (thermomixer).
- Directly proceed to section 3.7.2.

### 3.4 Cell fixation and permeabilization [timing: ~30 minutes]

- Fix cells onto coverslips in freshly made 2% formaldehyde/1x PBS for 10 minutes at RT.
- Rinse twice in 1x PBS.
- Permeabilize cells in freshly made ice-cold 0.5% Triton-X/1x PBS on ice. Permeabilization solution must be supplemented with 2mM Ribonucleoside-Vanadyl Complex, as an RNase inhibitor, when performing a 3D RNA FISH experiment. Time of permeabilization depends on the cell type and the size of the cytoplasm. We are routinely using a 5-minute permeabilization for cells that harbor a small cytoplasm such as mESC, thymic T cells (double-negative (DN) and double positive (DP) T cells), naive B cells (from the bone marrow and/or the spleen), and bone marrow derived macrophages (BMDM). For cells that display enlarged nuclei and an accompanying increase in cytoplasm volume such as T helper (TH) cells (change in nucleus size through T cell activation is shown in **Figure 2C**), peritoneal thioglycolate-elicited macrophages (TEPMs) and megakaryocytes, up to 15-20 minutes may be necessary for efficient permeabilization. The concentration of Triton-X100 can also be reduced for milder incubations.
- Rinse the coverslips twice with 1x PBS when performing immunofluorescence experiments, or with 2x SSC when performing FISH experiments, in order to remove any residuals from the permeabilization buffer. Proceed to section 3.5 for immunofluorescence, section 3.6 for 3D RNA FISH or section 3.7 for 3D DNA FISH. At this step coverslips can alternatively be stored for later usage. For long-term storage, rinse coverslips once in fresh 70% ethanol then add 70% ethanol and store at -20°C for 4 weeks for 3D DNA FISH, or less than 2 weeks for 3D RNA FISH (longer time might result in a degradation of the RNA). Note that this long-term storage is not recommended for immunofluorescence, as 70% ethanol may destroy most of the epitopes.

### 3.5 Immunofluorescence [timing: ~4 hours]

- Following step 3.4.d, block unspecific epitopes by incubating the cells in blocking solution (1x PBS/5% BSA; see **Table 2**) for 30 minutes. If background signals are observed, then natural goat serum and Tween can be added in order to reduce unspecific signal (see alternative blocking solution, **Table 2**). When aiming for a 3D immuno-RNA FISH experiment, adding 0.4U/μl of RNAse inhibitor is highly recommended to preserve RNAs.
- Dilute the primary antibody in 50-100μl of blocking solution, drop it onto an RNAse free hybridization cover (for example a microscope slide or a single layer of parafilm) placed into a humid hybridization chamber (including paper towels soaked in water). Place the coverslip cells side down onto it and incubate for 1 hour at RT or overnight at 4°C. Length and temperature of incubation should be troubleshooted as they depend on the antibody and cell types. We successfully perform 1 hour incubation at RT for most of antibodies against histone modifications and variants, for structural proteins like Lamin B1/A/C, NUPc, 53BP1 but also for transcription factors such as DROSHA, SATB1, SPEN etc.
- Wash 3 times for 5 minutes in 1x PBS under agitation (200-300rpm). In order to remove the background signal. 0.2% BSA and 0.1% Tween can be added.
- Incubate cells with the secondary antibody for 30-45 minutes at RT in blocking solution, in a dark and humid chamber as fluorophores are light-sensitive. As for the primary antibody incubation an alternative blocking solution (**Table 2**) can also be used to improve the signal quality and reduce the background if necessary.
- Wash 3 times for 5 minutes in 1x PBS in the dark under agitation (200-300rpm).
- Post-fix the cells in 2% formaldehyde for 10 minutes at RT.
- Rinse twice in 2x SSC and proceed with step 3.6.a for 3D immuno-RNA FISH, step 3.7.a. for 3D immuno-DNA FISH, or step 3.7.3.d. for simple IF and mounting.

### 3.6 3D RNA fluorescent *in situ* hybridization (RNA FISH) [timing: 2 days]

- Coverslips are placed cells side down onto a drop of ~10-20µl of probe hybridization mix (see Section 3.3.2.1) on a microscope slide in a dark and humid chamber (containing paper towels soaked in sterile water). Hybridize cells overnight with the probe(s) at 37°C.
- The next day, flood the coverslip with 2xSSC, gently remove it with forceps from the slides and transfer it (cells side up) to a 6-well plate containing 50% formamide/2x SSC.
- Wash three times in 50% formamide/2x SSC (pH 7.0-7.4) for 5 minutes in the dark at 42°C, under agitation (200-300rpm).
- Wash three times in 2x SSC for 5 minutes in the dark at 42°C, under agitation (200-300rpm). If a 3D RNA FISH or a 3D immuno-RNA FISH is performed, proceed straight to the mounting step in 3.7.3.d.
- If you wish to perform a DNA FISH afterwards, post-fix the cells in 2% formaldehyde for 10 minutes at RT.
- Rinse twice in 2x SSC and proceed with 3.7.a for 3D DNA FISH.

### 3.7 3D DNA fluorescent *in situ* hybridization (DNA FISH) [timing: 2 days]

- Permeabilize cells again in ice-cold 0.1M HCl/0.7% Triton-X for 10 minutes on ice.
- Rinse twice with 2x SSC.

For the DNA FISH part, we have successfully used 2 different protocols for probe and cell DNA denaturation, depending on the types of IF/FISH combinations and cell types. We describe both, although additional troubleshooting might be needed, in particular when the preservation of the 3D structure of the nuclei is disrupted (**Figure 2B**), the preservation of the IF and 3D RNA FISH signals is affected (see Problems 2-4), and the quality of the 3D DNA FISH signal is reduced (see Problems 5-7). Thus, perform either Option 1 or Option 2.

#### 3.7.1 Option 1: separate denaturation of probe(s) and cells

- Denature cells in 50% formamide/2x SSC (pH 7.0-7.4) at 80°C for 20-40 minutes. The incubation time should be optimized for each cell type. We usually use a 30-minute incubation (carefully transfer a 6-well plate floating in a water bath set at 80°C) for mESC and developing T lymphocytes (DN and DP cells). For larger cell types, incubation time should be further optimized (e.g., 40 minutes for murine helper T cells and macrophages).
- Rinse 3 times in ice-cold 2x SSC, to stop denaturation.
- Carefully drop the probes onto a glass microscope slide (prepared as in section 3.3.2.1) and place the coverslip cells side down onto it.
- Proceed to overnight hybridization at 37-42°C in a dark and humid chamber.

#### 3.7.2 Option 2: simultaneous denaturation of probe(s) and cells

- Carefully dispense the probe(s) (prepared as in section 3.3.2.2.) onto a glass microscope slide and gently transfer the coverslip cells side down onto it.
- Denature simultaneously probe(s) and cells for 2 to 5 minutes at 75°C on a hot metal plate in the dark. The incubation time should be optimized for each cell type. We usually use a 3-minute incubation for developing T lymphocytes (DN, DP cells, CD4^+^ and B cells), 4 minutes for mESC while for the differentiated T helper cells a denaturation of 5 minutes is required.
- Proceed to overnight hybridization at 37-42°C in a dark and humid chamber.

#### 3.7.3 Post-hybridization washes and mounting

- Next day, flood the coverslip with 50% formamide/2x SSC and gently transfer it with forceps (cells side up) to a fresh 6-well plate containing 50% formamide/2x SSC.
- Wash three times in 50% formamide/2x SSC (pH 7.0-7.4) for 5 minutes in the dark, at 42°C under agitation (200-300rpm).
- Wash three times in 2x SSC for 5 minutes in the dark, at 42°C under agitation (200-300rpm).
- Counterstain nuclear DNA with 1μg/ml DAPI for 2 minutes in the dark, rinse in 2x SSC and H_2_O. Coverslips are then mounted (cells side down) on a glass microscope slide. Slides can be kept at 4°C for short term or at -20°C for long term storage.

### 3.8 Image acquisition [timing: 1 day onwards]

**Figure 3:**
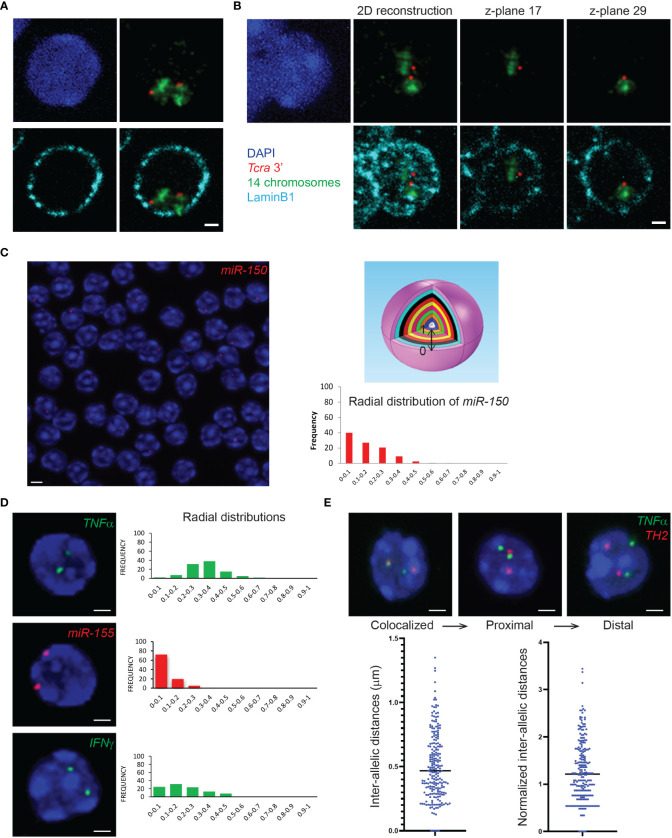
3D DNA FISH or 3D immuno-DNA FISH and gene nuclear distribution within the 3D nuclear space. **(A)** Representative single z-plane images of 3D immuno-DNA FISH in mouse DP T cells, depicting the T cell receptor locus *Tcra* (BAC clone RP23-255N13 labelled in Atto-dUTP 550, red) embedded in its corresponding chromosome territory (detected with XMP-14 whole chromosome paint (MetaSystems), green) and located close to the nuclear Lamina (LaminB1 antibody indirectly labeled with Alexa-680, cyan). **(B)** 2D image reconstruction and individual z-planes of 3D immuno-DNA FISH in DP T cells. The 2D reconstruction shows an artefactual proximity of the 2 *Tcra* alleles that are, in fact, located far from each other, in different z-planes. **(C)** (Left) Representative single z-plane image of 3D DNA FISH in thymocytes. The genomic *miR-150* locus of interest (BAC clone RP24-246G23) is detected using an Atto-dUTP 550 labeled DNA probe (red). (Top right) Schematic sphere depicting normalization of the gene positioning within the nucleus as 10 isocentric clusters of equal volume (ND=0-0.1 to ND=0.9-1), where ND=0-0.1 and ND=0.9-1 represent nuclear periphery and nuclear center, respectively. (Bottom right) Bar graph of the radial nuclear distribution of *miR-150* normalized to the nuclear radius, using the Volocity 3D image software. **(D)** Representative single z-plane images of 3D DNA FISH in T cells. The loci of interest are detected using an Atto-dUTP 550 labeled DNA probe for the non-coding *miR-155* locus (BAC clone RP24-278G19, red) and Atto-dUTP 488 labeled DNA probes for the coding *TNFα* (BAC clone RP23-446C22, green*)* and *IFNγ* (BAC clone RP24-352N22, green) loci. Bar graphs show the radial distribution of the genes of interest (*TNFα*, *miR-155* and *IFNγ*) in the nucleus. **(E)** Representative single z-plane images of 3D DNA FISH detecting *TNFα* (BAC clone RP23-446C22, green) and *TH2* (BAC clone B182, red) loci in CD4^+^ cells. Bar graphs represent the distribution of the intra-allelic distances between the *TNFα* and *TH2* alleles, before and after normalization to the nuclear radius. Nuclei are counterstained with DAPI (blue). Scale bar = 2 µm.

3D immuno-RNA/DNA FISH experiments can be scanned as 3D stacks with an appropriate microscope system with a dedicated camera for fluorescence imaging. A crucial aspect of the image acquisition for immuno-FISH experiments is the choice of the microscope depending on signal intensities. Confocal laser scanning microscopy requires strong and bright FISH signals but provides images with higher quality and reduced noise, while the wide-field epi-fluorescence microscopy is recommended for low intensity signals but may need additional deconvolution steps prior to analyses. We have successfully used SP5 and SP8 confocal microscopes (Leica), IXPlore spinning disk/wide-field dual module microscope (Olympus), Apotome (Zeiss) and Delta Vision microscope (Applied precision). Another important parameter is the acquisition of multiple z-planes in order to reconstruct nuclei in three dimensions for accurate 3D-analyses of distances. The distance between consecutive planes for 3D stacking depends on the resolution of the microscope. We usually use 0.2-0.3μm z-steps with either a 60X or a 100X magnification objective. **Figure 3A** depicts an example of a single plane of a z-stack (scanned with a 0.2μm step) showing the two *Tcra* alleles embedded in their corresponding chromosome territory in DP T cells. On the contrary **Figure 3B** shows a nucleus with the two *Tcra* alleles found in different planes, indicating that within the 3D nuclear space they are located far from each other. It is important to note that flattening the entire z-stack in order to build a 2D image reconstruction, would show an artefactual proximity between the two *Tcra* alleles (**Figure 3B** “2D reconstruction”).

### 3.9 Image analyses [timing: 2-3 days onwards]

**Figure 4:**
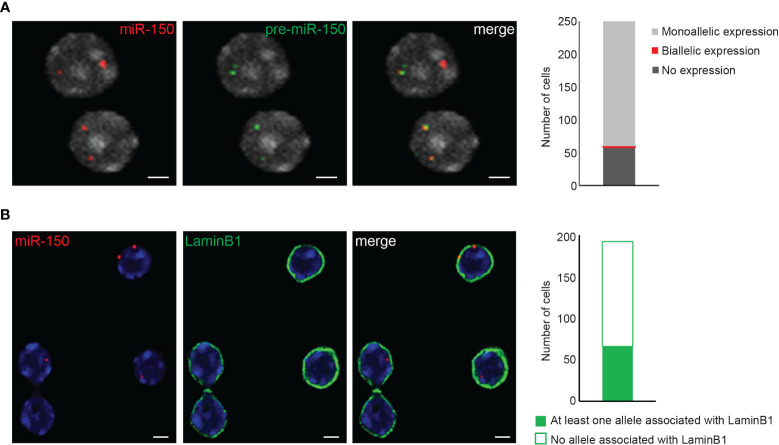
3D RNA/DNA FISH and 3D immuno-DNA FISH. **(A)** Example of confocal single z-plane images of 3D RNA/DNA FISH showing the colocalization of the nascent pri-miR-150 (BAC clone RP24-246G23, green) along with its corresponding gene locus (red) in mouse thymocytes. Bar graph represents the number of nuclei harboring monoallelic or biallelic expression of *miR-150*. Total number of nuclei analyzed is n=243. Scale bar 2µm. **(B)** Representative confocal single z-plane images of 3D immuno-DNA FISH, indicating the colocalization of *miR-150* alleles (red) with the nuclear Lamina (LaminB1 antibody indirectly labeled with labeled with Alexa-488 fluorochrome (green)) in thymocytes. Bar graph shows the number of nuclei with at least one allele associated with the LaminB1 signal. Total number of nuclei analyzed is n=193. Nuclei are counterstained with DAPI (gray in **(A)**, blue in **(B)**. Scale bar = 2 µm.

The images obtained from the 3D immuno-RNA/DNA FISH experiments can be analyzed by various available software packages. We have successfully used ImageJ/FIJI (free software from the NIH), Imaris (Oxford instruments) and Volocity (PerkinElmer) softwares. In order to map the exact location of a gene within the nucleus, in our lab we have been extensively using the Volocity 3D Visualization module which has the ability to visualize, identify, quantify and separate objects between them (ex. nuclei and IF or FISH signals). Based on the 3D reconstructed images, Volocity 3D Visualization module can determine the volume of the cells, define the exact location of the objects of interest (subnuclear compartments, genes/alleles etc.), and distinguish the distances between the center of the fluorescent signal (alleles) and the edge of the nucleus as defined by DAPI staining (or IF signal like LaminB1). The absolute distances between either the two alleles or between the alleles and nuclear compartments are normalized by the cell radius or the cell volume. In order to analyze and visualize object distances, we usually cluster them in 10 isocentric shells of equal volume within a 3D reconstructed cell nucleus (0-0.1 to 0.9-1), where 0 and 1 are determined as nuclear periphery and nuclear center respectively (**Figure 3C**). In order to obtain experimental reproducibility only nuclei displaying similar range of volumes between different biological replicates are usually analyzed in order to avoid analysis of either swollen or dehydrated cell nuclei. Moreover, analyzing a minimum of 100-150 nuclei with two visible alleles and an intact 3D architecture is usually recommended. Furthermore, the analysis should be conducted in a “blind manner” and, if possible, by two independent investigators. Colocalization between alleles or nuclear compartments can be determined either by a partial or a complete overlap between signals. Signal overlap can occur either between two nuclear compartments such as chromosome territory and nuclear Lamina (**Figures 3A, B**), between different genes or alleles and/or their corresponding primary transcripts (**Figures 3E**, **4A**) or between alleles and a nuclear compartment such as the nuclear Lamina (**Figure 4B**).

## 4 Results/expected outcomes

The chromatin of eukaryotic cells is spatially organized, and the nucleus is functionally and highly sub-compartmentalized. During most of the cell’s lifespan, chromosomes occupy distinct regions in the nucleus. Within each chromosome territory, chromatin conformation is dynamically condensed and decondensed in a transcriptional activation dependent manner (69, 70). Chromatin organization is considered as a determinant of the T cell lineage commitment and identity, with several groups documenting the global changes in chromatin accessibility, epigenomic marks and gene repositioning during T cell development and differentiation (6, 19, 22, 26, 71–73). Mapping of the 3D architecture can be achieved either through sequence-based profiling technologies such as the 3C-derived methodologies or the imaging-based research approaches (3D DNA FISH) that allow the visualization of the genes of interest at the single-allele and single-cell level. 3D DNA FISH can be used to detect compaction/decompaction of chromatin during different developmental stages and to visualize the changes of the localization of genes of interest within the 3D nuclear space, with their radial positioning or their positioning/repositioning relative to nuclear functional compartments such as the nucleolus or the nuclear Lamina.

The 3D immuno-RNA/DNA FISH protocol presented here allows the visualization of several genomic loci (3D DNA FISH) and their expression (nascent transcripts; 3D RNA FISH) relative to their chromosome territories (CT DNA FISH) and/or to nuclear proteins (IF), that can be simultaneously detected within the 3D nuclear space in a variety of immune cell types. In the current protocol we tried to include not only all the major steps that lead to a successful experiment but to also point out the critical steps during the process and discuss the possible limitations of the methodology. Using the aforementioned detailed protocol for 3D immuno-RNA/DNA FISH, we have been able to visualize coding and non-coding genes and their transcript in ESC and during the development and differentiation of T cells. 3D DNA FISH can provide the radial position of a gene within the nucleus (ie. central versus peripheral) but also the dynamics of its changes in location between different developmental stages of the T compartment (**Figure 3C**). Hybridization for three different genomic loci revealed 3 different types of distributions within the 3D nuclear space in thymocytes (**Figure 3D**). The *miR-155* locus exhibited a preferential peripheral location, with the most external shell cluster (ND=0-0.1) including more than 70% of all *miR-155* alleles, while on the contrary, the *TNFα* locus, showed a more internal location, with the majority of alleles occupying a more internal shell cluster (ND=0.3-0.4) (**Figure 3D**). On the other hand, the *IFNγ* locus revealed a broader distribution within the nucleus, with the clusters ND 0-0.1, ND 0.1-0.2, ND 0.2-0.3, ND 0.3-0.4 and ND 0.4-0.5 containing 24.5%, 31.3%, 23.3%, 13% and 8% of total alleles respectively. 3D DNA FISH can also indicate colocalization between genes especially when these genes are being coregulated or found colocalized, or in proximity to functional subnuclear compartments such as transcription factories (74). DNA FISH hybridization for the *TNFα* and *TH2* loci in CD4^+^ cells, revealed that 2% of the alleles of these two loci were found colocalized (ie. either adjacent or overlapping pixels) while 8% of the *TNFα* and *TH2* alleles were found in proximity (defined as a distance of <0.6μm) in a total of 200 nuclei analyzed (**Figure 3E**).

**Figure 5:**
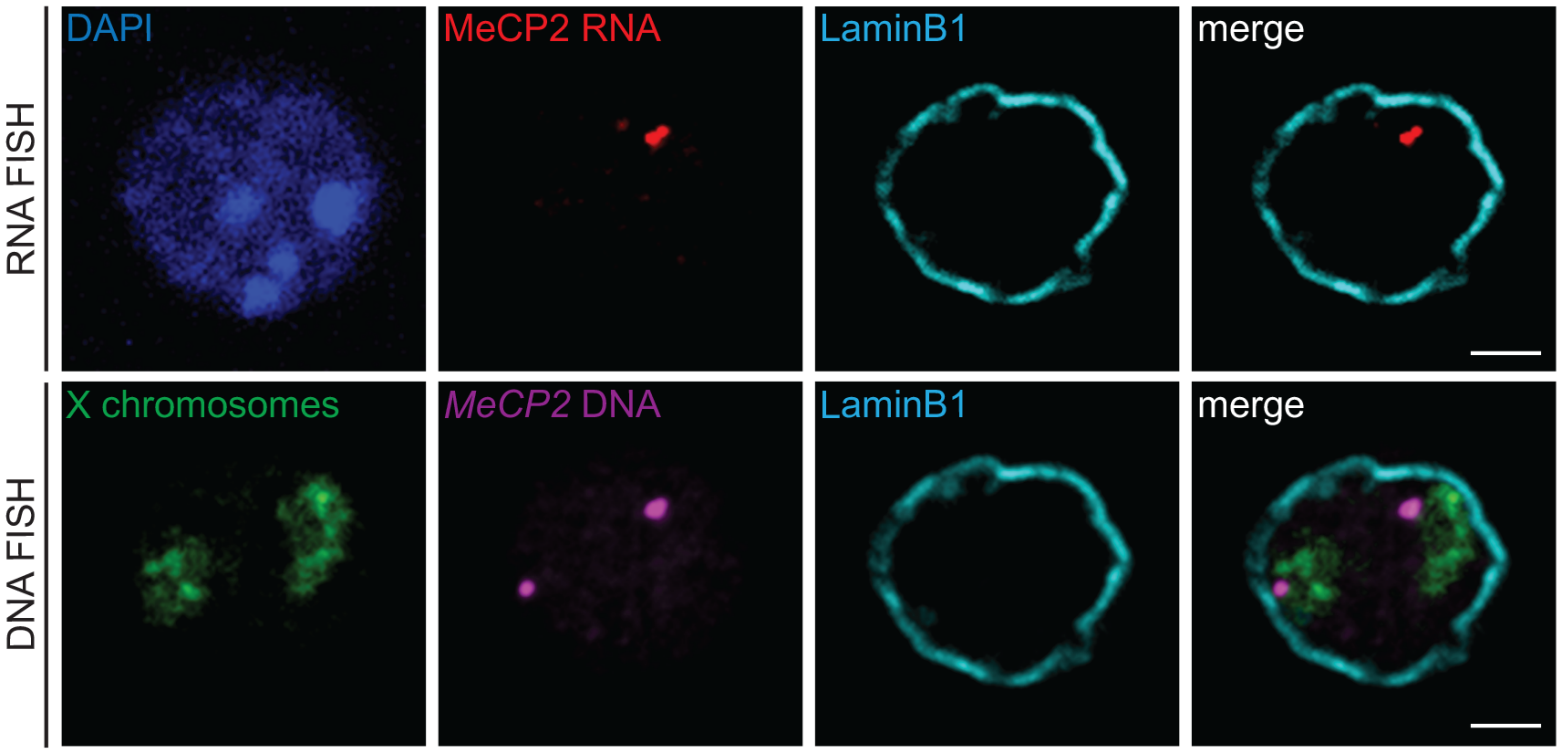
3D immuno-RNA/DNA FISH. Representative single z-plane images of a sequential 3D immuno-RNA/DNA FISH experiment in mouse embryonic stem cells. First, the 3D immuno-RNA FISH part detected MeCP2 primary transcripts (Atto-dUTP 550 labeled WI1-1229O10 fosmid probe, red) with the architectural LaminB1 protein (LaminB1 antibody indirectly labeled with Alexa-680 fluorochrome, cyan). After image recording, the 3D immuno-DNA FISH part was performed for the *Mecp2* genomic locus (same probe as for the RNA FISH part, magenta), the X chromosome territories (detected with XMP-14 whole chromosome paint (MetaSystems), green) and the Lamin B1 protein (cyan). Nuclei were counterstained with DAPI (blue). Scale bar = 2 µm.

3D RNA/DNA FISH enables the simultaneous detection of the primary transcripts and their corresponding transcribing gene locus as depicted in **Figure 4A** for the *miR-150* gene. Several conclusions can be drawn from such experiment that are complementary to results from qPCR or bulk RNA-seq. Indeed, with 3D RNA/DNA FISH, the percent of cells within a cell population expressing a gene can be directly assessed as well as whether a gene is expressed in a mono- of biallelic manner. Our RNA FISH analysis in thymocytes revealed that *miR-150* is mostly expressed in a monoallelic manner with less than 2% of the total cells displaying biallelic expression (**Figure 4A**). Complementary 3D immuno-DNA FISH experiments in thymocytes reported that 30% of the *miR-150* alleles are colocalized with Lamin B1, a component of the repressive compartment of the peripheral nuclear Lamina (**Figure 4B**). We also described here a sequential protocol (see Problem 3) which enables the visualization of the primary transcript, its corresponding genomic locus, its chromosome territory and a nuclear protein of interest. We were able to visualize the *Mecp2* DNA locus, its primary mRNA transcript, its corresponding X chromosome territory and the structural protein Lamin B1 in mESC as shown in **Figure 5**.

One of the most important considerations for a successful 3D RNA/DNA FISH experiment, is the ability to preserve the 3D structure of the nuclei. The crucial steps that might affect the 3D structure are fixation, permeabilization, hybridization, denaturation, and the overall handling of the samples during a hybridization experiment. The temperatures used during denaturation and hybridization, should be kept constant without any fluctuation since this might interfere with the signal background (see problems 2 and 8). If any of the FISH parameters is not properly optimized for each cell type, it might affect nuclear integrity (dehydrated or swollen) and causes changes in the nuclei size, leading to false measurements/analyses of the inter or intra allelic distances and radial positioning.

We recommend that each experiment is performed with three independent biological replicates where the nuclear volumes do not display major differences. The diameter of naïve lymphocytes and ESC varies between 4-8 μm, while the activated lymphocytes, macrophages and MEFs display a diameter between 8-15μm. If, for example, during two different DNA FISH sample preparation, ESC nuclei show a median of 4μm and 10 μm for the first and second experiment respectively, that means that the second experiment cannot be trusted since the nuclei represent a bigger volume, meaning that the cell preparation of the second replicate needs to be repeated. If the nuclei display a disrupted 3D structure, as shown in **Figure 2B**, further optimization as explained below (problem 1) should be performed. 3D DNA FISH is a time-consuming process, and a major consideration of the protocol is the preparation of the coverslips with sufficient cells that can be analyzed. Sparse cells will require a higher amount of time to scan whilst confluent cells have the tendency to attach with each other (**Figure 2A**). During analysis, overlap of the cells will interfere with the identification of nuclei as single objects in order to calculate their volume and/or diameter that will be necessary for distance normalization.

Further optimization steps and quality control details for 3D immuno-RNA/DNA FISH experiments are presented below:

**Problem 1:** Disrupted 3D nuclear structure

Potential solution: DAPI staining or immunostaining for structural perinuclear protein such as LaminB1 or LaminA/C might be used as a control for examining the 3D preserved structure of the nuclei. If the DAPI staining reveals disrupted nuclei with fuzzy edges and/or abnormal morphology (chromatin extruding outside the nuclear periphery) (**Figure 2B**), this might be due to under-fixation and/or over-permeabilization and/or over-denaturation during the cell preparation. A possible solution could be the increase of formaldehyde from 2% to 4% during cell fixation (more than 4% of formaldehyde fixation may prevent hybridization due to over-crosslinking). The over-permeabilization can be solved by decreasing the length of permeabilization or the concentration of Triton-X used (0.1-0.5% Triton-X for 3-15min). Other protocols have also reported the use of 50% glycerol and snap-freezing of the cells during fixation in order to maintain intact 3D nuclei (75). If the disrupted 3D nuclear structure is due to the denaturation procedure, then decreasing the time of denaturation may also help. Alternatively, if the two different denaturation options described in this protocol do not work, a third denaturation option consisting of a 30-minute incubation in 1.9M HCl at RT followed by 3 quick washes in ice-cold 2x SSC before overnight hybridization can alternatively be used (26, 27, 59).

**Problem 2**: Absence of immunofluorescence signal

Potential solution: No IF signal detection might suggest that the FISH procedure (overnight hybridization at 37-42°C and/or DNA denaturation) is either interfering with the antibody-epitope binding and/or disrupting the fluorophores of the secondary antibody. In this case the RNA/DNA FISH part may be performed before the IF part for highly expressed proteins such as RNA Pol II, LaminB1, NUPs, BRDA, Med12, and CTCF. If this solution is not working or in case of a lowly expressed nuclear protein of interest, an alternative sequential protocol is described in Problem 3.

**Problem 3**: Absence of immunofluorescence and 3D RNA FISH signals

Potential solution: If DNA FISH signal is detected but there is no RNA FISH and/or IF signals, this may indicate that the denaturation procedure during the DNA FISH part is disrupting the previous stainings (destroying the fluorophores). A possible solution here is troubleshooting of the denaturation process (already discussed in Problems 1 and 2). Alternatively, in our lab we are routinely using a sequential protocol, where we perform first the IF and 3D RNA FISH parts, take images under a microscope capable of recording the coordinates of the fields of view, and then proceed to the DNA FISH part (**Figure 5**) (26). In this case, cells are dropped onto glass slides instead of coverslips. Briefly, after mounting the slides and recording the first IF/3D RNA FISH part of the experiment, slides are unmounted by incubating them in a 50ml falcon containing 4X SSC-02% Tween 20 at 42°C for 5-10 minutes (two or three times), until the coverslips fall from the slide. After an additional wash in 2x SCC, the 3D DNA FISH can be performed as described in the section 3.7.

**Problem 4:** Weak RNA FISH signal

Potential solution: For weak RNA FISH signals of small genes, a signal amplification may be conducted using the TSA Biotin System following the manufacturer’s instructions, with few changes (60). Briefly, after the overnight hybridization, cell-spotted coverslips are rinsed with 2x, 1x and 4x SCC buffer for 3 minutes each, for the non-hybridized biotinylated probe residuals to be removed. Cells are then blocked for 30 minutes with TNB blocking buffer (100 mM Tris-HCl pH 7.5, 150 mM NaCl, 0.5% blocking reagent provided with the kit) in a dark and humid chamber and then incubated with streptavidin (SA), conjugated with horseradish peroxidase (HRP) in a dilution of 1/200 in TNB buffer for additional 30 minutes at RT. Cells are washed twice with TNT buffer (100 mM Tris-HCl pH 7.5, 150 mM NaCl, 0.05% Tween 20) for 3 minutes at RT. Cells are then incubated for 10 minutes at RT with biotinylated tyramide in a dilution of 1/50 in amplification diluent (supplied by the kit) and rinsed twice with TNT buffer for 3 minutes. For the visualization of the amplified RNA signal, cells are incubated with fluorophore-conjugated SA-488/555/647 for 30 minutes at RT, washed twice with TNT buffer and once with 1x PBS for 3 minutes each, and mounted as described in section 3.7.3.

**Problem 5**: Absence of RNA and/or DNA FISH signal

Potential solution: If no FISH signal is detected, this may be due to the over-fixation or under-permeabilization of the nuclei. Decreasing formaldehyde fixation (both time and/or concentration) and/or increasing the length of the permeabilization or concentration of Triton-X100 can be troubleshooted. Additionally, in order to facilitate probe hybridization, cells may be permeabilized before fixation, using either 0.5% Triton X-100/1x PBS or a cytoplasm removal buffer (cytoskeleton buffer (CSK) containing 100 mM NaCl, 300 mM sucrose, 3 mM MgCl2, 10 mM PIPES pH6.8, 1 mM EGTA, 0.5% Triton X-100) supplemented with 2 mM ribonucleoside-vanadyl complex for 3D RNA FISH. CSK treatment may vary between different cell types (3 minutes are suggested for mESC, thymocytes, naïve T and B cells while for activated helper T cells, MEFs and macrophages, 5-6 minutes of CSK treatment are recommended). Upon cytoplasm removal, cells are then fixed with 2-4% formaldehyde as described in section 3.4.

**Problem 6**: Unspecific or faint FISH signals with background

Potential solution: Increasing the stringency of post hybridization washes, using 0.2 to 1x SSC instead of 2x SSC and/or increasing the temperature of the washes (up to 45°C) may reduce the background. Background signal may also be reduced when the overnight hybridization temperature is increased up to 42°C. Another possible solution could be the increase of the amount of Cot-1 DNA or the length of pre-annealing incubation time of the FISH probes before hybridization.

**Problem 7:** Absence of DNA FISH signal

Potential solution: If no DNA FISH signal can be visualized, this may mean that the genomic DNA is not adequately denatured. Increasing the denaturation time and trying the alternative denaturation procedure (as described in Problem 1) is recommended. DNA FISH probes may also require some optimization (increasing the amount of probe used per each hybridization and/or increasing Cot-1 DNA competition to decrease background). Although RNA FISH probes can cover small sequences, DNA FISH probes spanning less than 5-10kb on the genomic DNA may also result in very faint signal. For the visualization of small genomic regions, performing high-resolution imaging may be recommended.

**Problem 8:** Signals accumulated at the perinuclear region

Potential solution: Accumulation of IF and/or FISH signals around the nuclear periphery is often due to inadequate permeabilization of the nuclei, preventing the antibody and/or probes to reach their corresponding targets within the nucleus. In this case, either longer permeabilization or CSK treatment (see Problem 5) may be used.

## 5 Discussion

Even though recent high-throughput genome-wide molecular methodologies (including HiC, Hi-ChIP, DamID) (15) have revolutionized the field with the possibility to dissect simultaneously many gene interactions and associations to chromatin or nuclear factors using populations of cells, 3D immuno-FISH still remains a powerful and complementary methodology. Indeed, it allows the simultaneous identification of few fluorescently labeled target sequences (DNA and/or RNA) and the analysis of their spatial arrangement and positioning with respect to nuclear compartments at the single nucleus resolution level. Advances on imaging systems have been made for the past 20 years (including resolution, speed of imaging, number of simultaneous fluorophores being imaged, and/or automation of the analyses), yet one limitation of 3D IF-FISH remains the ability of mapping/screening simultaneously multiple loci due to the fluorochromes/chromatic restrictions set by spectral overlap. Usually, five colors are needed for a full 3D immuno- RNA/DNA FISH (including gene and chromosome territory) experiment. In order to avoid the spectrum limitations, when targeting a higher number of gene loci, sequential imaging could be applied (see problem 3).

Furthermore, the spectrum limitation can also be resolved using the oligopaint barcoded probes, which provide a higher flexibility in the number of targets identified each time (47). Establishing any kind of *in situ* hybridization is not always straightforward protocol and may require extensive troubleshooting. Moreover, the analysis of the deconvoluted images can also be considered as a long process. Even though nowadays most of the softwares used for the analysis of the 3D RNA/DNA FISH experiments provide the option of an automated identification of the FISH signals and the calculation of distances at the level of individual nuclei, a visual inspection and optimization by the “human eye” is still necessary. Despite all the limitations and shortcomings described here, when compared with 3C derived assays, FISH still remains a very useful methodology when single cell spatial arrangement is required. Moreover, FISH validation is also required as a complementary analysis or reconfirmation assay on the ligation-based chromatin conformation capture technologies that are based on populations of cells. Another major limitation of the *in-situ* methodology is the fixation of the cells which does not allow visualization of the dynamics of chromatin interactions and transcription or gene relocalization during development. The 3D DNA FISH methodology defines the exact position of the gene with the 3D nuclear space, though without capturing the kinetics and the order of the repositioning events during the activation of cell differentiation. Notwithstanding the technical advances in 3D DNA FISH, the chromatin organization field is being redirected into studying genome structures in four-dimensions (4D) through live cell imaging, with only few groups so far successfully reporting 4D live cell imaging of genomic loci (53, 76, 77). Further technical advances and a higher resolution in the 4D live cell imaging direction, will provide more details on the dynamics and kinetics of enhancer-promoter communication of cell type specific genes and will open new avenues to study causality between the 4D genome and gene regulation during development and immune responses.

## Data availability statement

The raw data supporting the conclusions of this article will be made available by the authors, without undue reservation.

## Ethics statement

The animal study was reviewed and approved by Greek ethical committee of animal experimentation, approved by the General Directorate of Veterinary Services, Region Crete (license number: EL91BIO-02).

## Author contributions

ES, CGS and JC conceived and optimized the protocols in the ESC and immune and hematopoietic cell subtypes. Troubleshooting of the differentiated TH1 and TH2 cells and images provided in **Figures 3C-E**, **4** have been generated by ES in the laboratory of CGS. The rest of the images have been generated exclusively in the JC laboratory. CGS and JC supervised the studies. ES and JC wrote, revised and edited the manuscript with help from CGS. All authors contributed to the article and approved the submitted version.
